# Caffeine Inhibits Oxidative Stress- and Low Dose Endotoxemia-Induced Senescence—Role of Thioredoxin-1

**DOI:** 10.3390/antiox12061244

**Published:** 2023-06-09

**Authors:** Dennis Merk, Jan Greulich, Annika Vierkant, Fiona Cox, Olaf Eckermann, Florian von Ameln, Nadine Dyballa-Rukes, Joachim Altschmied, Niloofar Ale-Agha, Philipp Jakobs, Judith Haendeler

**Affiliations:** 1 Cardiovascular Degeneration, Haendeler Group, Clinical Chemistry and Laboratory Diagnostics, Medical Faculty, University Hospital, Heinrich-Heine University Duesseldorf, 40225 Duesseldorf, Germany; dennis.merk@hhu.de (D.M.); fiona.cox@hhu.de (F.C.); olaf.eckermann@hhu.de (O.E.); nadine.dyballa@hhu.de (N.D.-R.); 2 Cardiovascular Degeneration, Altschmied Group, Clinical Chemistry and Laboratory Diagnostics, Medical Faculty, University Hospital, Heinrich-Heine University Duesseldorf, 40225 Duesseldorf, Germany; jan.greulich@hhu.de (J.G.); annika.vierkant@hhu.de (A.V.); florian.ameln@hhu.de (F.v.A.); joalt001@hhu.de (J.A.); 3 Institute for Translational Pharmacology, Medical Faculty, University Hospital, Heinrich-Heine University Duesseldorf, 40225 Duesseldorf, Germany; 4 CARID, Cardiovascular Research Institute Düsseldorf, Medical Faculty, University Hospital, Heinrich-Heine University Duesseldorf, 40225 Duesseldorf, Germany

**Keywords:** caffeine, endothelial cells (EC), lipopolysaccharide, senescence, Thioredoxin-1

## Abstract

The maintenance of Thioredoxin-1 (Trx-1) levels, and thus of cellular redox homeostasis, is vital for endothelial cells (ECs) to prevent senescence induction. One hallmark of EC functionality, their migratory capacity, which depends on intact mitochondria, is reduced in senescence. Caffeine improves the migratory capacity and mitochondrial functionality of ECs. However, the impact of caffeine on EC senescence has never been investigated. Moreover, a high-fat diet, which can induce EC senescence, results in approximately 1 ng/mL lipopolysaccharide (LPS) in the blood. Therefore, we investigated if low dose endotoxemia induces EC senescence and concomitantly reduces Trx-1 levels, and if caffeine prevents or even reverses senescence. We show that caffeine precludes H_2_O_2_-triggered senescence induction by maintaining endothelial NO synthase (eNOS) levels and preventing the elevation of p21. Notably, 1 ng/mL LPS also increases p21 levels and reduces eNOS and Trx-1 amounts. These effects are completely blocked by co-treatment with caffeine. This prevention of senescence induction is similarly accomplished by the permanent expression of mitochondrial p27, a downstream effector of caffeine. Most importantly, after senescence induction by LPS, a single bolus of caffeine inhibits the increase in p21. This treatment also blocks Trx-1 degradation, suggesting that the reversion of senescence is intimately associated with a normalized redox balance.

## 1. Introduction

During the aging process and in a variety of diseases, there is an increase in reactive oxygen species (ROS, e.g., O_2_^−^ and H_2_O_2_), which can lead to senescent endothelial cells (ECs) in humans [1]. Thioredoxin-1 (Trx-1) is a ubiquitously expressed oxidoreductase, which reduces ROS in concert with peroxiredoxins. Trx-1 also interacts with several proteins in different compartments of the cell and thereby modulates their functions [2,3]. In ECs, Trx-1 is one of the most important anti-oxidative proteins [4,5]. In a previous study, we demonstrated in an oxidative-stress-induced senescence model (using H_2_O_2_) that the amount of Trx-1 protein is decreased in those senescent ECs and that the levels of the ROS-generating enzyme NADPH oxidase 4 are elevated, suggesting that disturbed redox homeostasis is linked to senescence induction. Along these lines, senescence induction could be blocked by the permanent expression of Trx-1 [6], demonstrating a causal relationship between the loss of Trx-1 and the appearance of cellular senescence.

Moreover, we were able to show that—in addition to ROS—100 mg/dL low-density lipoprotein (LDL) as well as ultrafine carbon nanoparticles also induce senescence in primary ECs [7].

One important feature of EC functionality is their ability to migrate, which is compromised in senescent ECs [7,8]. We found that the migratory capacity of ECs depends on intact mitochondria [8,9]. Moreover, short-term treatment with caffeine in concentrations up to 50 µM, which corresponds to 4–6 cups of coffee per day [9,10], improves the migratory capacity of ECs [9]. Interestingly, positive effects of caffeine in these doses were also shown in several cohort studies, in which an association was found between coffee consumption and a reduced mortality rate for several diseases affecting the cardiovascular system, most of which are associated with endothelial dysfunction. In these studies, the number of deaths from heart and respiratory diseases, stroke, and type II diabetes correlated negatively with coffee consumption, and the mortality risk was reduced by consumption of 4–6 cups a day compared to lower intake [11]. Altogether, these data suggest that caffeine could sustain EC functionality by counteracting processes leading to their dysfunction, which is a hallmark of senescent cells.

Recently, we unraveled the molecular mechanism explaining the protective role of caffeine. We demonstrated that caffeine increased the translocation of p27, a cell cycle inhibitor, into the mitochondria, leading to improved mitochondrial functionality. In p27-deficient mice, electron transport chain activity was reduced compared to their wild-type littermates and could not be improved with caffeine. This clearly demonstrated that the caffeine effects are mediated through an increase in mitochondrial p27. In addition, we found that caffeine was able to increase the functionality of mitochondria isolated from 24-month-old mice to the level of 6-month-old animals [12] and thus reverse the aging-associated functional decline of these organelles.

So far, we have shown that the maintenance of Trx-1 protein levels, and thus cellular redox homeostasis, has important beneficial effects on ECs by preventing senescence induction. Moreover, caffeine treatment results in functional improvements. However, the effects of caffeine on EC senescence have never been investigated. Furthermore, a high-fat diet has been shown to result in approximately 1 ng/mL lipopolysaccharide (LPS) in the blood [13]. Our finding that a simulated high-fat diet with elevated LDL levels induces EC senescence [7] suggests that low dose LPS might have a similar effect, although this has so far not been analyzed. Therefore, we investigated if low dose endotoxemia can induce EC senescence and concomitantly reduce Trx-1 levels, and if caffeine can counteract induction or even reverse senescence.

## 2. Materials and Methods

### 2.1. Cell Culture

Primary human ECs were supplied by LONZA (Cologne, Germany), and human embryonic kidney cells (HEK293T) were supplied by Invitrogen (Darmstadt, Germany). ECs and HEK293T were cultured as previously described [14,15]. In detail, ECs were cultured in endothelial basal medium supplemented with 50 ng/mL amphotericin B, 1 μg/mL hydrocortisone, 50 μg/mL gentamicin, 12 μg/mL bovine brain extract, 10 ng/mL epidermal growth factor (LONZA, Cologne, Germany), and 10% fetal bovine serum until the third passage was reached. Cells were grown for at least 20 h after detachment with trypsin. HEK293T cells were cultured in DMEM GlutaMAX™ (Invitrogen, Darmstadt, Germany) supplemented with 10% heat-inactivated fetal bovine serum and 1% penicillin/streptomycin.

### 2.2. Lentiviral Production and Transduction

VSV-G pseudotyped lentiviral particles were generated as previously described [6]. In detail, HEK293T cells were co-transfected with a transfer vector and expression vectors for the VSV-G envelope protein and lentiviral Gag/Pol, using the Calcium Phosphate Transfection Kit (Invitrogen, Darmstadt, Germany) according to the manufacturer’s instructions. Viral particles containing supernatant were collected over three days, then filtered through a 0.45 μm PVDF membrane (Millipore, Burlington, MA, USA) and concentrated using Vivacell 100 ultrafiltration units with a PES membrane and a 100,000 MW cutoff (Sartorius, Goettingen, Germany). Concentrated virus particles were stored in aliquots at −80 °C. Viral titer was determined with the QuickTiter Lentivirus Titer Kit (Lentivirus-Associated HIV p24) (Cell Biolabs, San Diego, CA, USA). ECs were transduced with lentiviral expression vectors for mitochondrial p27 [12] with a multiplicity of infection of 50. The cells were washed 3 times 24 h after transduction.

### 2.3. Transient Transfection of ECs

Transient transfections of ECs with plasmid DNA were performed using Effectene (Qiagen, Hilden, Germany). In detail, ECs were transfected on 6 cm culture dishes with 2.5 µg plasmid DNA, 20 µL enhancer, and 25 µL Effectene in 150 µL buffer, with the subsequent addition of 1 mL cell culture medium.

### 2.4. Migration Assay

Migration was quantitated with a scratch wound assay, as previously described [12]. In detail, wounds were set by scraping cell monolayers with a sterile disposable rubber policeman. For that purpose, ECs were cultivated on 6 cm dishes, which were labeled with a trace line before setting the wound. After applying them to the injury, non-attached cells were removed by gentle washing with culture medium. Quantification of EC migration from the edge of the injured monolayer was performed by staining the cells with 500 ng/mL 4′,6-diamidino-2-phenylindole (DAPI) (Carl Roth, Karlsruhe, Germany) in PBS after the cells were fixed with 4% paraformaldehyde for 15 min at room temperature. Microscopic pictures were taken using an Axio Observer Z (Zeiss, Jena, Germany) using a 200× magnification. The cells which had invaded the wound from the trace line were automatically counted using the particle analysis feature of Image J 1.52a after watershed separation of overlapping nuclei.

### 2.5. Immunoblotting

Immunoblotting was performed as previously described [16]. In detail, cells were detached from the culture surface with a rubber policeman, centrifuged at 800× *g,* and washed twice with ice-cold PBS. After final centrifugation at 800× *g* and removal of the supernatant, cells were resuspended in radioimmunoprecipitation assay (RIPA) buffer (50 mM Tris-HCl pH 8.0, 150 mM NaCl, 1% (*v*/*v*) IGEPAL^®^-CA630, 0.1% (*w*/*v*) SDS, and 0.5% (*w*/*v*) Na-Deoxycholate) supplemented with 1/100 volume of a protease inhibitor cocktail and phosphatase inhibitor cocktail (Bimake, Munich, Germany) and lysed for 30 min on ice. Then, the lysates were centrifuged at 18,000× *g* and 4 °C for 15 min and the supernatant was transferred to a fresh tube. Proteins were separated by sodium dodecyl sulfate–polyacrylamide gel electrophoresis according to standard procedures. After the transfer of the proteins onto polyvinylidene difluoride membranes and blocking with 5% milk powder in TBS (200 mM Tris-HCl pH 8.0, 300 mM NaCl, and 100 mM KCl) with 0.1% (*v*/*v*) Tween-20 (TBS-T) for 1 h at room temperature, membranes were incubated with antibodies directed against GAPDH (1:50.000), Trx-1 (1:500), eNOS (1:500) (all three from Abcam, Cambridge, UK), p21 (1:500), and Src (1:1000) (both from Cell Signaling Technologies, Frankfurt, Germany). Antibodies were incubated overnight at 4 °C. The following day, membranes were washed three times with TBS-T and incubated with secondary antibodies coupled to horseradish peroxidase (ECL anti-rabbit or anti-mouse IgG, horseradish-peroxidase-linked whole antibody (from sheep) (1:5000) (Cytiva, Marlborough, MA, USA). Detection was performed using ECL substrate (Cytiva, Marlborough, MA, USA) and X-ray films. Semi-quantitative analyses were performed on scanned X-ray films using Image J [17].

### 2.6. Immunostaining of ECs

Immunostaining of ECs was performed as previously described [12]. In detail, cells were fixed with 4% formaldehyde for 15 min and were blocked and permeabilized for 15 min at room temperature with 3% (*v*/*v*) normal goat serum (Sigma-Aldrich, Deisenhofen, Germany) diluted in PBS containing 0.3% (*v*/*v*) Triton X-100. Afterward, the cells were incubated with primary antibodies against a myc-tag or p21 (all from Cell Signaling Technologies, Frankfurt, Germany), each 1:100, overnight at 4 °C. The antibody against TIM23 (BD BioSciences, Heidelberg, Germany) was diluted 1:150 and incubated overnight at 4 °C. Subsequently, cells were washed three times with PBS and incubated with an Alexa Fluor^®^ 594 coupled anti-mouse or anti-rabbit secondary antibody (1:500) (Santa Cruz Biotechnology, Heidelberg, Germany) for 1 h at room temperature. Nuclei were counterstained with DAPI (500 ng/mL) (Carl Roth, Karlsruhe, Germany) in PBS for 5 min at room temperature and the cells were mounted with ProLong™ Diamond Antifade Mountant (Invitrogen, Darmstadt, Germany). Images were taken using Zeiss microscopes (Axio Observer Z or Axio Imager M2, Zeiss, Jena, Germany) using a 400× or 200× magnification. Pixel intensities were measured with Image J [17].

### 2.7. Measurement of Intracellular ROS by Fluorescence Microscopy

ROS levels were quantitatively assessed using dihydroethidine (DHE) and MitoSOX™-Red, and mitochondria were co-stained with MitoTracker^®^ Green FM (all purchased from Molecular Probes, Eugene, OR, USA). Cells were washed with endothelial basal medium and incubated with either 10 µM DHE or 5 µM MitoSOX™-Red combined with 100 nM MitoTracker^®^ Green FM for 30 min at 37 °C. Afterward, cells were washed twice with endothelial basal medium, and images were taken with an Axio Observer Z (Zeiss, Jena, Germany) using a 200× magnification. Fluorescence intensity was calculated with Image J and normalized to the cell number.

### 2.8. Total Cellular RNA Isolation

Total cellular RNA was isolated as previously described [18]. In detail, TRIzol (Thermo Fisher Scientific, Dreieich, Germany) was used to extract total RNA from ECs according to the manufacturer’s instructions. Further purification of RNA was achieved by using the RNeasy^®^ Mini kit (Qiagen, Hilden, Germany) and concentrations were measured using a NanoDropTM 2000c (Thermo Fisher Scientific, Dreieich, Germany). RNA integrity and purity were analyzed by agarose gel electrophoresis.

### 2.9. cDNA Synthesis

cDNA was synthesized as previously described [18]. In detail, total cellular RNA was reverse transcribed using the QuantiTect Reverse Transcription kit (Qiagen, Hilden Germany) according to the manufacturer’s instructions. For the verification of the expression of transgenes after lentiviral transduction, cDNA was synthesized with or, as a control for residual genomic DNA containing proviral genomes, without reverse transcriptase.

### 2.10. Polymerase Chain Reaction (PCR)

Endpoint PCRs were performed with MyTaq^TM^ HS DNA Polymerase (Biocat, Heidelberg, Germany) and primers for RPL32 (hm RPL32 Ex02 for1 5′-GTGAAGCCCAAGATCGTCAA-3′ and hm RPL32 Ex03 rev1 5′-TTGTTGCACATCAGCAGCAC-3′) and mito p27 (hCDKN1B Ex01 for1 5′-GGTTAGCGGAGCAATGCG-3′ and myc-tag rev2 5′-TCCTCTTCTGAGATGAGTTTTTGTTC-3′) according to manufacturer’s recommen-dations in a Bio-Rad T100 Thermal Cycler (BioRad, Feldkirchen Germany). The reaction products were visualized on standard agarose gels.

Semi-quantitative real-time PCRs were used to determine the relative transcript levels of Trx-1 with corresponding primers (hm TXN1 Ex01 for1 5′-TGGTGAAGCAGATCGAGAGC-3′ and hm TXN1 Ex03/04 rev1 5′-ACATCCTGACAGTCATCCACAT-3′), cDNA as a template, and the primaQUANT 2x qPCR SYBR-Green-MasterMix (Steinbrenner, Wiesenbach, Germany) in a Rotor-Gene Q instrument (Qiagen, Hilden, Germany). Relative expression was calculated by the ΔC_t_ method with RPL32 as a reference [19].

### 2.11. Statistics

The number of experiments (n) represents independent biological replicates. The data presented are mean ± SEM. Normal distribution was confirmed by the Shapiro–Wilk test and homogeneity of variances (from means) was verified by Levene’s test. Since all data sets represented normal distribution, the multiple comparisons were performed using one-way ANOVA with the post hoc Tukey LSD test. Pairwise comparisons were performed with a paired Student’s *t*-test on the raw data.

## 3. Results

### 3.1. Caffeine Prevents Stress-Induced Senescence in Endothelial Cells

In previous work, we demonstrated that stress-induced senescence of ECs entails an increase in the cell cycle inhibitor p21 and a decrease in the levels of eNOS, the enzyme constitutively producing NO to preserve the NO bioavailability and functionality of ECs. Moreover, the amount of Trx-1, which is essential for cellular redox homeostasis, was decreased and the migratory capacity of ECs was compromised. Lentiviral re-expression of Trx-1 in this model inhibited senescence induction, underscoring the importance of Trx-1 [6]. We also demonstrated that 100 mg/dL LDL induces EC senescence, reduces mitochondrial functionality, including ATP production, and impairs migration [8]. Interestingly, caffeine induces EC migration [9], enhances mitochondrial functionality in old mouse hearts, and improves the outcomes after myocardial infarction in prediabetic mice [12]. Thus, one could speculate that caffeine could be beneficial in stress-induced senescence in ECs.

**Figure 1:**
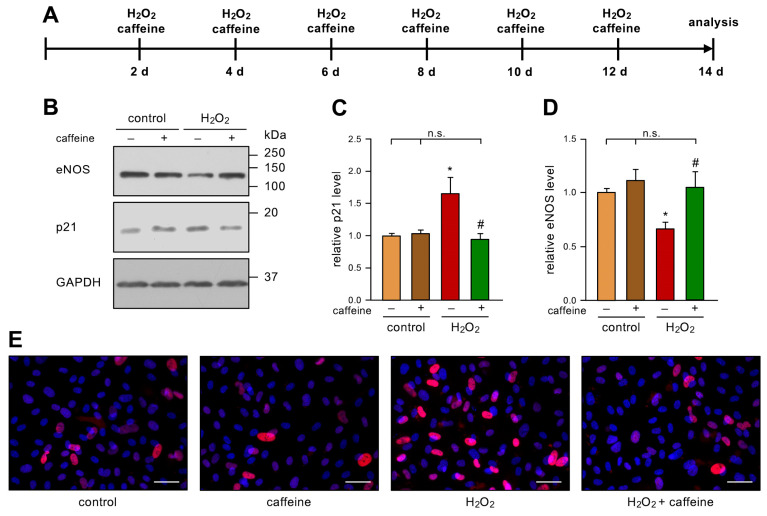
Caffeine prevents H_2_O_2_-induced senescence. (**A**–**E**) ECs were treated with 50 µM H_2_O_2_ and 10 µM caffeine every second day for two weeks. (**A**) Schematic representation of the treatment. (**B**–**D**) p21 and eNOS were detected by immunoblot, and GAPDH served as loading control. The untreated controls and the H_2_O_2_-treated group received caffeine (+) or not (−), as indicated. (**B**) Representative immunoblots for p21 (middle panel), eNOS (upper panel), and GAPDH (lower panel). (**C**,**D**) Semi-quantitative analysis of relative amounts of p21 (**C**) and eNOS (**D**) (data are mean ± SEM, n = 5, * *p* < 0.05 vs. control, ^#^ *p* < 0.05 vs. H_2_O_2_ without caffeine, n.s. = not significant, and one-way ANOVA with post hoc Tukey LSD test). (**E**) p21 was detected by fluorescence microscopy. ECs were stained with an anti-p21 antibody (red) and nuclei were counterstained with DAPI (blue). Shown is a representative immunostaining (scale bar = 50 µm).

To investigate the role of caffeine in stress-induced senescence in ECs, we used an established model [6]. In detail, we treated primary human ECs with 50 µM H_2_O_2_ and 10 µM caffeine every two days for two weeks (Figure 1A). As expected and previously published [6], H_2_O_2_-treatment-induced senescence in ECs was shown by an increase in p21. Interestingly, this effect was completely blocked by the co-treatment, while caffeine alone did not affect the p21 levels (Figure 1B,C,E). As a second marker of EC senescence, we investigated the levels of eNOS in this model. Repetitive treatment with H_2_O_2_ led to a significant decrease in eNOS protein levels, as observed previously [6]. However, the additional treatment with caffeine prevented the H_2_O_2_-induced loss of eNOS. As for p21, caffeine alone had no effect on eNOS protein levels (Figure 1B,D).

### 3.2. Caffeine Counteracts Low Dose Endotoxemia-Induced Senescence in Endothelial Cells

**Figure 2:**
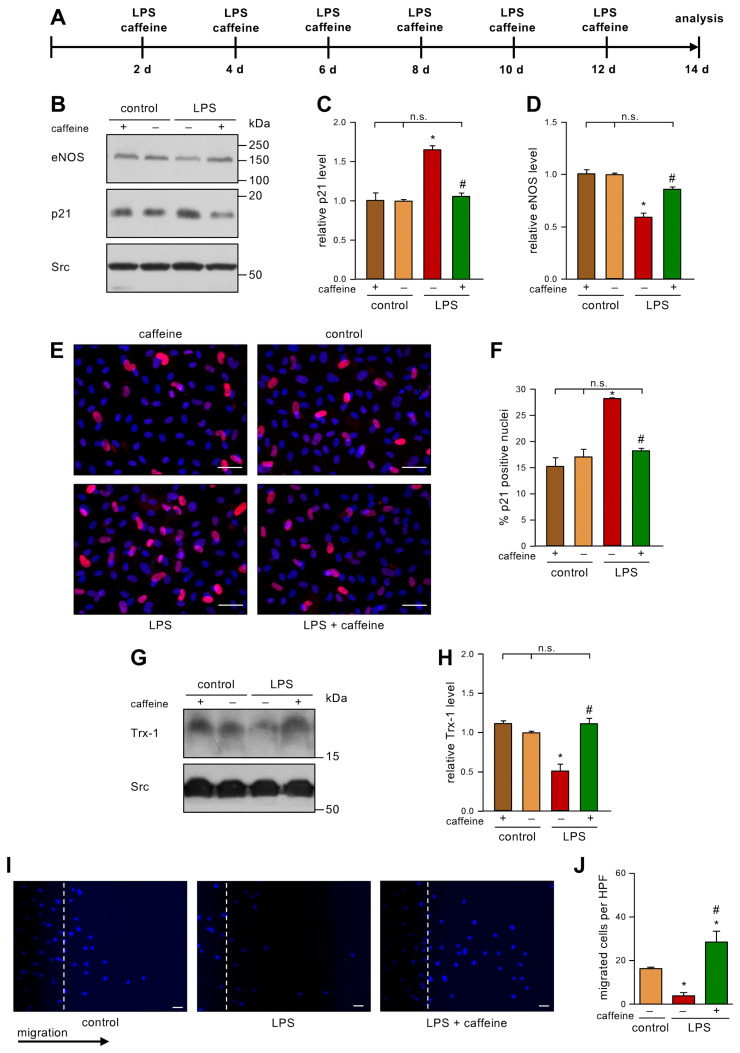
Caffeine counteracts LPS-induced senescence, maintains Trx-1 levels, and increases migratory capacity. (**A**–**J**) ECs were treated with 1 ng/mL detoxified (control) or active LPS (LPS) and 10 µM caffeine every second day for two weeks. (**A**) Schematic representation of the treatment. (**B**–**J**) The controls treated with detoxified LPS and the LPS-treated group received caffeine (+) or not (−), as indicated. (**B**–**D**) p21 and eNOS were detected by immunoblot, and Src served as a loading control. (**B**) Representative immunoblots for p21 (middle panel), eNOS (upper panel), and Src (lower panel). (**C**,**D**) Semi-quantitative analysis of the relative amounts of p21 (**C**) and eNOS (**D**) (data are mean ± SEM, n = 4, *p < 0.05 vs. control, ^#^ *p* < 0.05 vs. LPS without caffeine, n.s. = not significant, and one-way ANOVA with post hoc Tukey LSD test). (**E**,**F**) p21 was detected by fluorescence microscopy. Cells were stained with an anti-p21 antibody (red) and nuclei were counterstained with DAPI (blue). (**E**) Representative immunostainings (scale bar = 50 μm). (**F**) Image J analyses of the percentage of p21 positive nuclei (data are mean ± SEM, n = 4, * *p* < 0.05 vs. control, ^#^ *p* < 0.05 vs. LPS without caffeine, n.s. = not significant, and one-way ANOVA with post hoc Tukey LSD test). (**G**,**H**) Trx-1 was detected by immunoblot and Src served as a loading control. (**G**) Representative immunoblots for Trx-1 (upper panel) and Src (lower panel). (**H**) Semi-quantitative analysis of the relative amounts of Trx-1 (data are mean ± SEM, n = 4, * *p* < 0.05 vs. control, ^#^ *p* < 0.05 vs. LPS without caffeine, n.s. = not significant, and one-way ANOVA with post hoc Tukey LSD test). (**I**,**J**) Migratory capacity was detected by scratch wound assays. Cell nuclei were stained with DAPI. (**I**) Representative DAPI staining (scale bar = 50 µm). (**J**) Semi-quantitative analysis of migrated cells per high-power field (HPF) (data are mean ± SEM, n = 4, * *p* < 0.05 vs. control, ^#^ *p* < 0.05 vs. LPS without caffeine, and one-way ANOVA with post hoc Tukey LSD test).

After having shown that caffeine is able to inhibit H_2_O_2_-induced senescence in ECs, we wanted to use a potentially more relevant inducer of EC senescence. In our hands, senescence could also be induced with 100 mg/dL LDL [7]. As it had been demonstrated that a high-fat diet, which leads to diabetes and insulin resistance, results in up to 1 ng/mL LPS in the blood [13], we next investigated if low dose endotoxemia can also induce senescence in ECs, and if this could be prevented with caffeine. Therefore, we treated human ECs repetitively with 1 ng/mL LPS every second day for a total of two weeks and co-incubated those cells with 10 µM caffeine (Figure 2A). As a control, we used LPS from the same *E. coli* serotype that had been partially delipidated by alkaline hydrolysis. This detoxified LPS has an endotoxin level about 10,000 times lower than the parent LPS. In this setting, we demonstrate here for the first time that 1 ng/mL LPS is able to induce a senescent phenotype in ECs, as shown by an increase in p21 (Figure 2B,C,E,F). Moreover, we found that this dose of LPS induces ROS (Supplementary Figure S1). Co-treatment with caffeine prevented this upregulation of p21 (Figure 2B,C,E,F), while caffeine alone had no effect. Next, we analyzed whether LPS also affects eNOS levels. Repetitive low doses of LPS reduced the amount of eNOS significantly (Figure 2B,D) and this effect was inhibited by caffeine. As before, caffeine alone had no effect on eNOS protein levels. Next, we determined the levels of Trx-1 as an indicator of the cellular redox status. LPS significantly reduced the amount of Trx-1 (Figure 2G,H) comparable to the reduction shown with H_2_O_2_ in our previous study [6]. On the mRNA level, neither H_2_O_2_ nor LPS affected Trx-1 (Supplementary Figure S2), indicating that low dose endotoxemia—like oxidative stress—induces the degradation of the protein. Interestingly, co-treatment with caffeine completely restored Trx-1 protein levels (Figure 2G,H). As a functional cellular read-out, we determined the migratory capacity of ECs under this low dose endotoxemia by performing scratch wound assays. The LPS treatment dramatically reduced migration, and this detrimental effect was blocked by caffeine treatment (Figure 2I,J). We had previously shown that caffeine enhances the import of p27 into mitochondria and that the pro-migratory effect of caffeine in ECs completely depends on mitochondrial p27 [12]. Thus, it was tempting to speculate that the protective effects of caffeine in LPS-induced senescence are mediated through mitochondrial p27.

### 3.3. Permanent Expression of Mitochondrial p27 in Endothelial Cells Inhibits Senescence Induction by Low dose Endotoxemia

**Figure 3:**
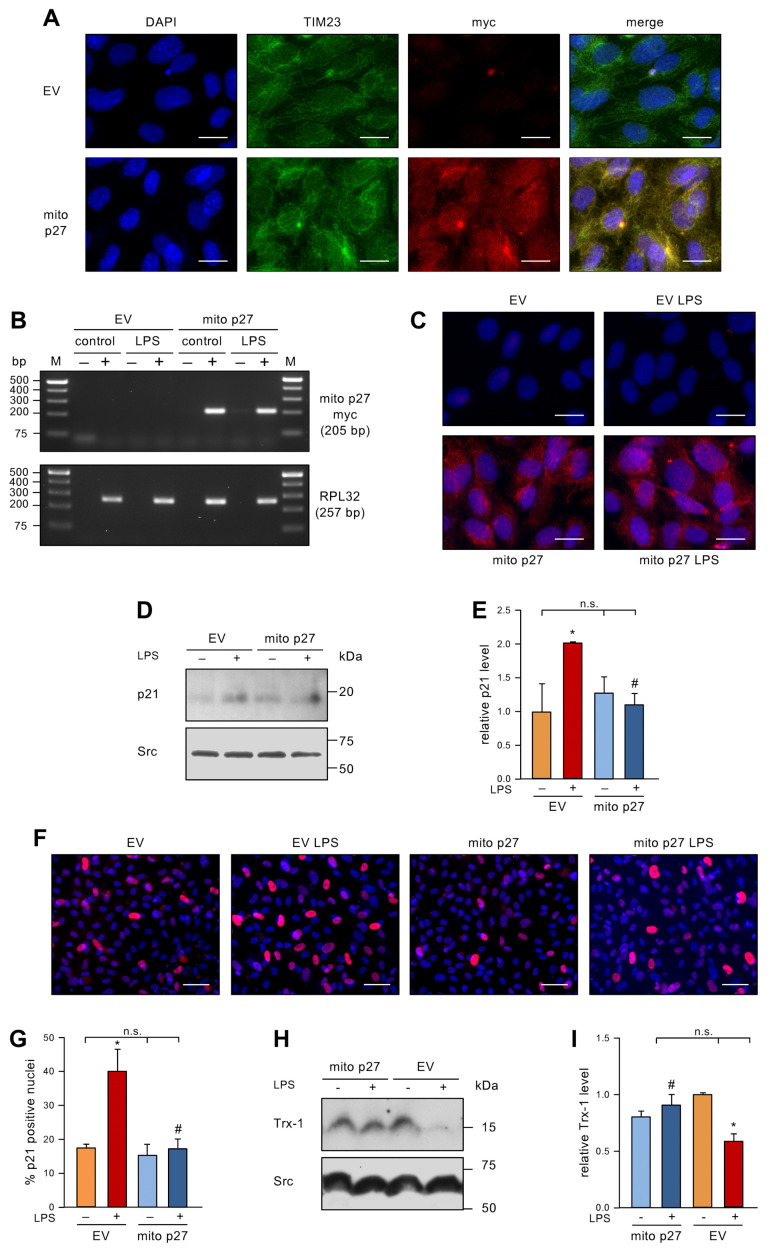
Mitochondrial p27 counteracts LPS-induced senescence and maintains Trx-1 levels. (**A**) ECs were transduced with a lentiviral expression vector for mitochondrially targeted p27 with a C-terminal myc epitope tag (mito p27) or a corresponding empty virus (EV) and after two weeks localization of mito p27 was examined by fluorescence microscopy. ECs were stained with antibodies directed against the myc-tag on mito p27 (red) and the translocase of inner mitochondrial membrane 23 (TIM23, green), and the nuclei were counterstained with DAPI (blue); shown are representative immunostainings (scale bar = 20 μm). (**B**–**I**) ECs were transduced with a lentiviral expression vector for mitochondrially targeted p27 with a C-terminal myc epitope tag (mito p27) or a corresponding empty virus (EV) and subsequently treated with 1 ng/mL detoxified (control/−) or active LPS (LPS/+) every second day for two weeks. (**B**) Expression of mito p27 was analyzed by reverse transcription polymerase chain reaction. For that purpose, RNA was isolated from the transduced cells, and cDNA was synthesized in the presence (+) or absence (−) of reverse transcriptase. Amplification was performed with primers specifically detecting the mito p27 myc transcript, while the housekeeping gene RPL32 served as control. Amplification products were resolved by agarose gel electrophoresis; the expected fragment sizes are specified, and the numbers on the left indicate DNA size markers (M). (**C**–**E**) p21 was detected by immunoblot and immunostaining. (**C**) ECs were stained with an anti-myc antibody (red), and nuclei were counterstained with DAPI (blue); shown are representative immunostainings (scale bar = 20 μm). (**D**,**E**) p21 was detected by immunoblot and Src served as a loading control. (**D**) Representative immunoblots for p21 (upper panel) and Src (lower panel). (**E**) Semi-quantitative analysis of relative amounts of p21 (data are mean ± SEM, n = 3, * *p* < 0.05 vs. EV without LPS, ^#^ *p* < 0.05 vs. EV + LPS, n.s. = not significant, and one-way ANOVA with post hoc Tukey LSD test). (**F**,**G**) p21 was detected by immunostaining and fluorescence microscopy. Cells were stained with an anti-p21 antibody (red), and nuclei were counterstained with DAPI (blue). (**F**) Representative immunostainings (scale bar = 50 μm). (**G**) Image J analyses of the percentage of p21 positive nuclei (data are mean ± SEM, n = 3, * *p* < 0.05 vs. EV without LPS, ^#^ *p* < 0.05 vs. EV + LPS, n.s. = not significant, and one-way ANOVA with post hoc Tukey LSD test). (**H**,**I**) Trx-1 was detected by immunoblot and Src served as a loading control. (**H**) Representative immunoblots for Trx-1 (upper panel) and Src (lower panel). (**I**) Semi-quantitative analysis of the relative amounts of Trx-1 (data are mean ± SEM, n = 3, * *p* < 0.05 vs. EV without LPS, ^#^ *p* < 0.05 vs. EV + LPS, n.s. = not significant, and one-way ANOVA with post hoc Tukey LSD test).

To investigate the impact of permanently elevated levels of mitochondrial p27 on LPS-induced EC senescence, we expressed mitochondrially-targeted p27 (mito p27) with a lentiviral vector [12] before repetitive treatment with LPS. After first confirming exclusive mitochondrial localization (Figure 3A), we showed that LPS treatment affected neither the transcript (Figure 3B) nor the protein levels (Figure 3C) of the lentivirally expressed mitochondrial p27. Next, we measured p21 protein levels by immunoblotting. In the control group transduced with an empty virus, the levels of p21 protein were elevated by LPS treatment. The transduction with the lentiviral mito p27 expression vector did not change p21 protein levels in cells not treated with LPS. Even more interestingly, p21 levels were not increased after repetitive LPS treatment when mitochondrial p27 levels were sustained (Figure 3D,E). Similar results were found by p21 immunostaining (Figure 3F,G). Along the same lines, Trx-1 protein levels were reduced by LPS treatment in the control group, yet transduction with a lentiviral mito p27 expression vector blocked LPS-induced Trx-1 degradation (Figure 3H,I). These data clearly demonstrate a protective effect of mitochondrial p27 as a downstream effector of caffeine in senescence induction in ECs, possibly by maintaining cellular redox homeostasis.

### 3.4. Caffeine Can Reverse Low dose endotoxemia-Induced Senescence in Endothelial Cells

**Figure 4:**
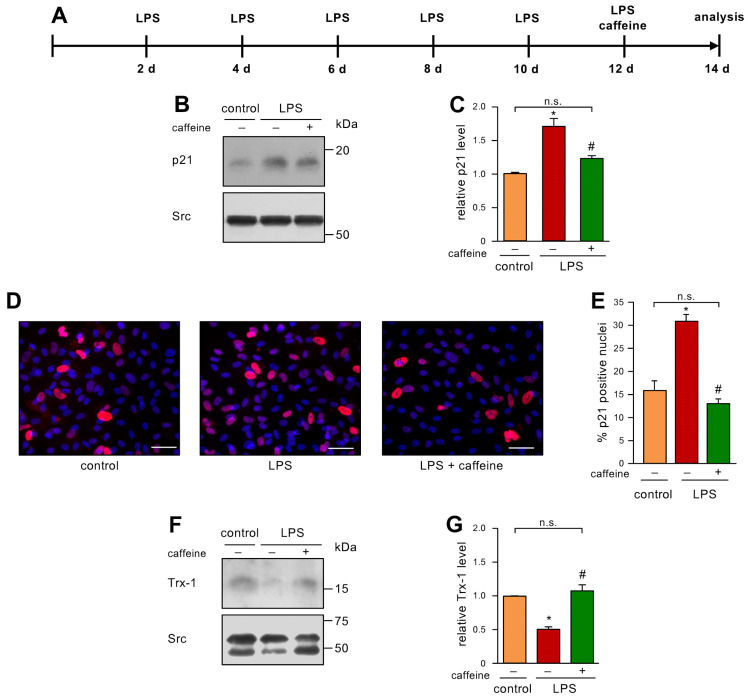
A single caffeine bolus reverses LPS-induced senescence and restores Trx-1 levels. (**A**–**G**) ECs were treated with 1 ng/mL detoxified (control) or active LPS (LPS) every second day for two weeks with a single bolus of 50 µM caffeine on the last treatment day. (**A**) Schematic representation of the treatment. (**B**–**G**) The LPS-treated group received caffeine (+) or not (−), as indicated. (**B**,**C**) p21 was detected by immunoblot and Src served as a loading control. (**B**) Representative immunoblots for p21 (upper panel) and Src (lower panel). (**C**) Semi-quantitative analysis of relative amounts of p21 (data are mean ± SEM, n = 5, * *p* < 0.05 vs. control, ^#^ *p* < 0.05 vs. LPS without caffeine, n.s. = not significant, and one-way ANOVA with post hoc Tukey LSD test). (**D**,**E**) p21 was detected by immunostaining and fluorescence microscopy. Cells were stained with an anti-p21 antibody (red), and nuclei were counterstained with DAPI (blue). (**D**) Representative immunostainings (scale bar = 50 μm). (**E**) Image J analyses of the percentage of p21 positive nuclei (data are mean ± SEM, n = 4, * *p* < 0.05 vs. control, ^#^ *p* < 0.05 vs. LPS without caffeine, n.s. = not significant, and one-way ANOVA with post hoc Tukey LSD test). (**F**,**G**) Trx-1 was detected by immunoblot and Src served as a loading control. (**F**) Representative immunoblots for Trx-1 (upper panel) and Src (lower panel). (**G**) Semi-quantitative analysis of relative amounts of Trx-1 (data are mean ± SEM, n = 4, * *p* < 0.05 vs. control, ^#^ *p* < 0.05 vs. LPS without caffeine, n.s. = not significant, and one-way ANOVA with post hoc Tukey LSD test).

Finally, we were interested in a therapeutic approach, to address the question of whether caffeine can reverse an already established senescent cellular phenotype. Therefore, we first induced senescence by LPS treatment as before and gave a single bolus of 50 µM caffeine after 12 days of LPS treatment (Figure 4A). This dose was chosen since we had already demonstrated that this concentration induces EC migration as a read-out for proper functionality. Moreover, 0.1% caffeine given with the drinking water resulted in a similar serum concentration in mice [9] and this regimen improved the respiratory chain activity in the heart mitochondria of 24-month-old mice to the level observed in mitochondria from 6-month-old animals [12]. As shown before, LPS treatment led to elevated p21 levels, and, interestingly, the single treatment with caffeine was sufficient to inhibit this increase in p21 (Figure 4B,C). These results were confirmed by immunostaining for p21 (Figure 4D,E). Similar to the results shown for the preventive treatment with caffeine (Figure 2G,H), the single treatment with caffeine blocked Trx-1 degradation induced by LPS (Figure 4F,G), suggesting that the reversion of senescence is intimately associated with a normalized redox balance.

## 4. Discussion

The major findings of the present study are the prevention and, even more importantly, the reversion of low dose endotoxemia-induced EC senescence by caffeine. Here we show for the first time that caffeine precludes senescence in ECs by preventing the loss of Trx-1 and eNOS, proteins vital for cellular redox homeostasis and NO bioavailability, respectively, and thereby, for the proper functionality of ECs. Moreover, we established a model for senescence induction by repetitive treatment with LPS in a low concentration. Indeed, in this low dose endotoxemia model, co-incubation with caffeine prevented the occurrence of senescence. Even more important, in a therapeutic setting, a single bolus of caffeine given on day 12 of senescence induction by LPS reversed senescence and the loss of Trx-1 protein.

Our data demonstrate that treatment of primary human ECs with 1 ng/mL LPS results in the onset of senescence and loss of EC functionality. This LPS concentration was chosen as several studies in mice and humans demonstrated that a high-fat diet leads to an increase in LPS levels in the blood. In mice, feeding a high-fat diet for 4 weeks increased the plasma LPS concentrations up to three times to a concentration of approximately 1.2 ng/mL [13]. Similarly, in healthy humans, a high fat, high carbohydrate meal resulted in elevated LPS levels in the blood when compared with humans consuming a high fiber and fruit meal [20]. The consumption of Western-type calorie rich diets combined with chronic overnutrition and a sedentary lifestyle represents a rising public health problem, as this often results in, for instance, type 2 diabetes and atherosclerosis. Given the fact that a high-fat diet leads to low dose endotoxemia, it is tempting to speculate that the senescent ECs found in human atherosclerotic plaques [1] are induced by increased LPS in the blood, as our findings here demonstrate that this low dose endotoxemia induces senescence in human ECs.

Senescence and aging are associated with mitochondrial dysfunction [21]. Here, we show that low dose endotoxemia reduces the migratory capacity of ECs. As previous studies demonstrated that the migration of ECs depends on intact mitochondria [9] and functional oxidative phosphorylation [8], one could assume that LPS treatment impairs electron transport chain activity and, thereby, diminishes migratory capacity. This would be in line with the findings of Deshpande et al. demonstrating that induction of senescence by constitutive active Rac1 results in increased mitochondrial ROS and decreased electron transport chain activity in ECs [22]. Similarly, we demonstrated that treatment of ECs with 100 mg/dL LDL results in senescence induction [7] and in a loss of mitochondrial ATP production [8]. Thus, mitochondrial functionality and EC senescence are intimately interwoven. Therefore, a substance that improves mitochondrial functions and, thus, endothelial functionality would be of great interest to prevent or delay endothelial dysfunction.

We have shown that caffeine in serum concentrations, which are reached after consumption of 4–6 cups of coffee, increases the migratory capacity of human ECs [9,10]. A few years ago, we unraveled the mechanisms underlying the promigratory effect of caffeine. We demonstrated that caffeine induces the translocation of p27 into the mitochondria and that the migratory capacity of ECs—also after stimulation with caffeine—is completely dependent on mitochondrial p27. Caffeine in the drinking water improved the respiratory chain activity in the hearts of wild-type mice, but not in p27-deficient littermates. More importantly, caffeine was able to increase the electron transport chain activity in the hearts of 24-month-old animals to the levels measured in adult, 6-month-old mice, suggesting an anti-aging effect of caffeine [12]. Here, we demonstrate that caffeine indeed inhibits senescence induction in ECs, possibly by improving mitochondrial functionality as the permanent expression of mitochondrially targeted p27 had the same effect. Caffeine also counteracted the loss of Trx-1 and, moreover, even when given in a therapeutic setting, i.e., after senescence induction by LPS, restored Trx-1 levels.

Since the maintenance of Trx-1 protein levels is important for preserving redox homeostasis as well as the ability to store NO in ECs, it is interesting to note that H_2_O_2_- as well as low dose LPS-induced senescence lead to the degradation of Trx-1 and increase in ROS ([6] and Supplementary Figure S1, respectively). Thus, one has to assume that disturbed redox homeostasis is a hallmark of senescence induction in ECs and thus, of endothelial dysfunction. Since caffeine is able to restore Trx-1 levels and inhibit senescence, it would be interesting to understand how caffeine is capable of doing so. The loss of Trx-1 in senescent ECs goes along with the elevated activity of cathepsin D [6], the major lysosomal protease, which is responsible for Trx-1 degradation [23,24]. As it is known that mitochondrial respiration controls lysosomal function and that impairment of respiration leads to an increase in the lysosomal compartment [25], it is tempting to speculate that caffeine reduces lysosomal activity. This is beyond the scope of this study but will be investigated in the future. However, it has to be noted that low dose endotoxemia, but also the treatment of ECs with 150 ng/mL LPS for 24 h does not change Trx-1 mRNA levels (Supplementary Figure S2 and [18]). Those data suggest that it is not the downregulation of Trx-1 expression, but rather enhanced degradation that leads to the decrease in Trx-1 levels in senescent ECs. Having shown that treatment with low dose LPS led to a degradation of Trx-1, and that caffeine, which improves mitochondrial functionality [12], counteracts Trx-1 degradation, it would be interesting to investigate if LPS and caffeine have similar effects on Thioredoxin-2, the mitochondrial Thioredoxin, in a future study.

## 5. Conclusions

In conclusions, the observation that caffeine interferes with senescence induction in ECs, which would lead to endothelial dysfunction that is observed in nearly all cardiovascular diseases [26], might at least partially explain the beneficial effects of moderate coffee consumption on mortality risk in elderly people [11,27].

## Data Availability

Data are contained within the article. All further information will be provided by the corresponding authors upon request.

## Supplementary Materials

The following supporting information can be downloaded at: https://www.mdpi.com/article/10.3390/antiox12061244/s1, Figure S1: Low dose endotoxemia increases ROS levels; Figure S2: Senescence induction does not change the Trx-1 transcript level.
