# Supplementary material for: Caffeine Inhibits Oxidative Stress- and Low Dose Endotoxemia-Induced Senescence—Role of Thioredoxin-1
Source: Antioxidants (Basel). 2023 Jun 9;12(6):1244. doi: 10.3390/antiox12061244 (PMC10294853; doi:10.3390/antiox12061244)
Supplement: Supplementary file 1 [file antioxidants-12-01244-s001.zip › antioxidants-2385005-supplementary.pdf]

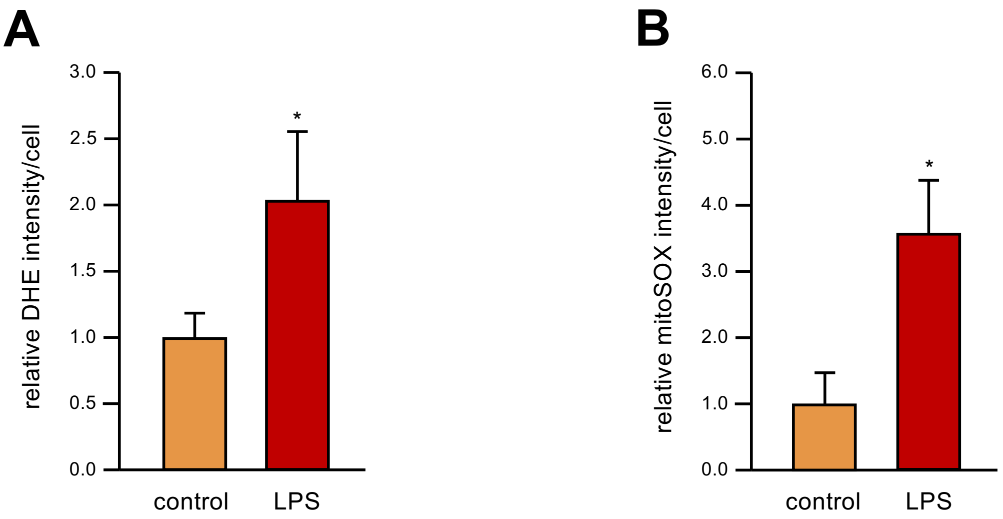

Supplementary figure S1: Low dose endotoxemia increases ROS levels. EC were treated with 1 ng/mL detoxified (control) or active LPS (LPS) every second day for two weeks. Rela-tive DHE (A) or mitoSOX (B) intensities were analyzed by Image J using total cell count for normalization (data are mean ± SEM, n = 3, *p < 0.05 vs control, two sided paired t-Test).

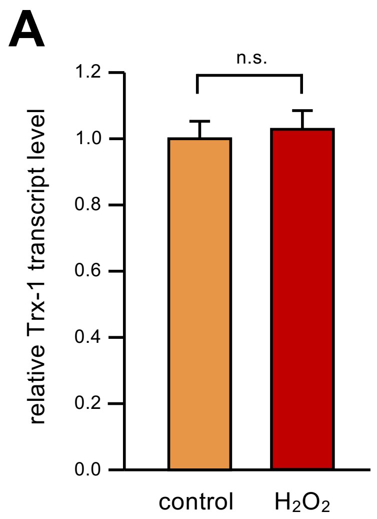

Supplementary figure S 2: Senescence induction does not change the Trx-1 transcript level. (A) EC were treated with 50 µM H_2O_2 every day for two weeks or left untreated (control). (B) In a low dose endotoxemia model, EC were treated with 1 ng/mL detoxified (control) or active LPS (LPS) every second day for two weeks. (A, B) Trx-1 transcript levels were ana-lyzed by semi-quantitative real-time PCR using RPL32 for normalization (data are mean ± SEM, n = 6, n.s. = not significant, two sided paired t-Test).
